# Low-frequency dual-frequency ultrasound-mediated microbubble cavitation for transdermal minoxidil delivery and hair growth enhancement

**DOI:** 10.1038/s41598-020-61328-0

**Published:** 2020-03-09

**Authors:** Ai-Ho Liao, Keng-Hsien Lin, Ho-Chiao Chuang, Chih-Hung Tsai, Yi-Chun Lin, Chih-Hung Wang, Cheng-Ping Shih, Hao-Li Liu

**Affiliations:** 1 Graduate Institute of Biomedical Engineering, National Taiwan University of Science and Technology, Taipei, 10607 Taiwan; 2 Department of Biomedical Engineering, National Defense Medical Center, Taipei, 11490 Taiwan; 3 Department of Mechanical Engineering, National Taipei University of Technology, Taipei, 10608 Taiwan; 4 Department of Electrical Engineering, Chang Gung University, Taoyuan, 33302 Taiwan; 5 Graduate Institute of Medical Sciences, National Defense Medical Center, Taipei, 11490 Taiwan; 6 Department of Otolaryngology–Head and Neck Surgery, Tri-Service General Hospital, National Defense Medical Center, Taipei, 11490 Taiwan; 7 Taichung Armed Forces General Hospital, Taichung, 41168 Taiwan; 8 Department of Neurosurgery, Chang Gung Memorial Hospital, Taoyuan, 33302 Taiwan

**Keywords:** Biophysics, Medical research

## Abstract

Ultrasound (US) has been found to rejuvenate and invigorate the hair follicles, increase the size of hair shafts, and promote new hair growth. Our present study found that dual-frequency US-mediated microbubble (MB) cavitation significantly enhanced minoxidil (Mx) delivery in both *in vitro* and *in vivo* models, while increasing the hair growth efficacy compared to single-frequency US sonication. The *in vitro* experiments showed that cavitation activity was enhanced more significantly during dual-frequency sonication than single-frequency sonication in higher concentration of MBs. The pigskin penetration depth in the group in which dual-frequency US was combined with MBs was 1.54 and 2.86 times greater than for single-frequency US combined with MBs and in the control group, respectively; the corresponding increases in the release rate of Mx at 18 hours in *in vitro* Franz-diffusion-cell experiments were 24.9% and 43.7%. During 21 days of treatment in C57BL/6J mice experiments, the growth rate at day 11 in the group in which dual-frequency US was combined with MBs increased by 2.07 times compared to single-frequency US combined with MBs. These results indicate that dual-frequency US-mediated MB cavitation can significantly increase both skin permeability and transdermal drug delivery. At the same US power density, hair growth was greater in the group with dual-frequency US plus MBs than in the group with single-frequency US plus MBs, without damaging the skin in mice.

## Introduction

Pressure waves generated by ultrasound (US) have been employed to enhance transdermal drug delivery (TDD) for many years^1^. In 2004, US at 20–100 kHz was approved by the United States FDA for the transdermal delivery of the local anesthetic lidocaine^2^. The shock waves contributing to the sonophoresis induced by US were hypothesized to structurally modify lipids of the stratum corneum to produce diffusion channels that allowed transdermal delivery^3–5^. The thermal effects of US also positively influence the drug diffusion coefficient and skin permeability coefficient, but might result in heating damage when administering thermally unstable transdermal drugs^6,7^.

US has recently been demonstrated to improve skin permeability and reduce thermal effects^8^. The most important mechanism for skin permeability enhancement induced by US is acoustic cavitation^9^. Inertial cavitation mediated by US has been previously shown to mediate much greater permeability enhancement of the skin surface to enhance the transdermal delivery of drugs and vaccines than stable cavitation^10–13^. A previous study demonstrated that US-assisted vaccine delivery in the presence of sub-micron cavitation nuclei resulted higher specific antibody levels compared to other transdermal methods^14^. Our previous studies demonstrated that combined treatment with US using optimal parameters and optimal conditions (size or concentration) of microbubbles (MBs) can increase skin permeability so as to enhance drug delivery without increasing temperatures or causing heating damage^15,16^. Minoxidil (Mx)-coated albumin-shelled MBs produced in a layer-by-layer manner combined with sonication by US energy in the water phase successively enhanced hair growth while shortening the treatment period^17^.

In comparison to single-frequency US sonication, multiple-frequency US excitation can markedly enhance the acoustic cavitation effect^18^. Dual-frequency US has been used to facilitate diagnoses via real-time superharmonic imaging^19^ and to enhance transdermal transport for therapeutic applications^20^. Dual-frequency US has involved combining US at a low frequency (ranging from 20 to 60 kHz) with high-frequency US (ranging from 1 to 3 MHz)^8^. A recent study indicated that dual-frequency US may be a useful treatment option for rosacea that is resistant to other treatments^21^. This method has emerged as a new treatment modality for improving inflammatory skin disorders. Moreover, in our previous study, dual-frequency US sonication generates more cavitation activity that may stimulate neural stem/progenitor cell differentiation and growth factor utilization, while not causing any damage^22^. The biological effect due to dual-frequency US exposure was found to be significantly higher than single-frequency US exposure, and we have shown that this correlated to the enhanced cavitation effect during dual-frequency US exposure. Some previous studies proposed the dual- and multiple-frequency US excitation generated from separate piezoelectric transducers^23–25^. However, our previous study demonstrated that dual- or multiple-frequency US wave irradiations are possible to be generated from a single crystal once the driving frequency matches the characteristic frequency of the transducer^26,27^, making the dual- or multiple-frequency US irradiation more feasible and bring opportunity for biomedical application such as transdermal drug delivery (TDD) enhancements.

MBs are contrast agents that are commonly used in contrast-enhanced US imaging of blood perfusion in organs, to detect the blood flow rate in the heart, and many other applications^28^. The effects of US-mediated albumin-shelled-MB cavitation in TDD were demonstrated in our recent study^15,16^. The following characteristics of MBs vary with their size: resonance frequency, expansion ratio, pressure threshold for inertial cavitation and fragmentation, translational velocity, and lifetime of stable oscillations^29^. The efficacy of US-mediated MB cavitation in increasing tissue permeability was dependent on the size distribution for the same acoustic pressure^15,30^. By utilizing nonisolated MBs with nonuniform sizes, dual-frequency US might be able to increase the efficacy of TDD. Therefore, in this study we aimed to demonstrate the potential of using dual-frequency US-mediated MB cavitation to enhance TDD.

Our previous study provided a new integrated TDD platform for monitoring the delivery of Mx to hair follicles by utilizing multifunctional albumin-shelled MBs and single-frequency US^17^. The present study is the first to investigate the potential of single- and dual-frequency US sonication for TDD and to enhance hair follicle growth both *in vitro* and *in vivo* with Mx or Mx mixed in MBs.

## Materials and Methods

### Preparation of albumin-shelled MBs

Albumin-shelled MBs were prepared in our laboratory according to the procedure used in our previous studies^15–17^. Briefly, albumin-shelled MBs were generated by 2 min of sonication (Branson Ultrasonics, Danbury, CT, USA) of 10 mL of a solution containing 132 mg of albumin (Octapharma, Vienna, Austria) and perfluoropropane (C3F8) gas in physiological saline (pH 7.4, 0.9% sodium chloride). The albumin-shelled MBs had a diameter of 1.02 ± 0.11 μm (mean ± SD) and a concentration of 1.40 × 10^8^ MBs/mL.

### Passive cavitation detection of single- and dual-frequency US-mediated MB cavitation

**Figure 1:**
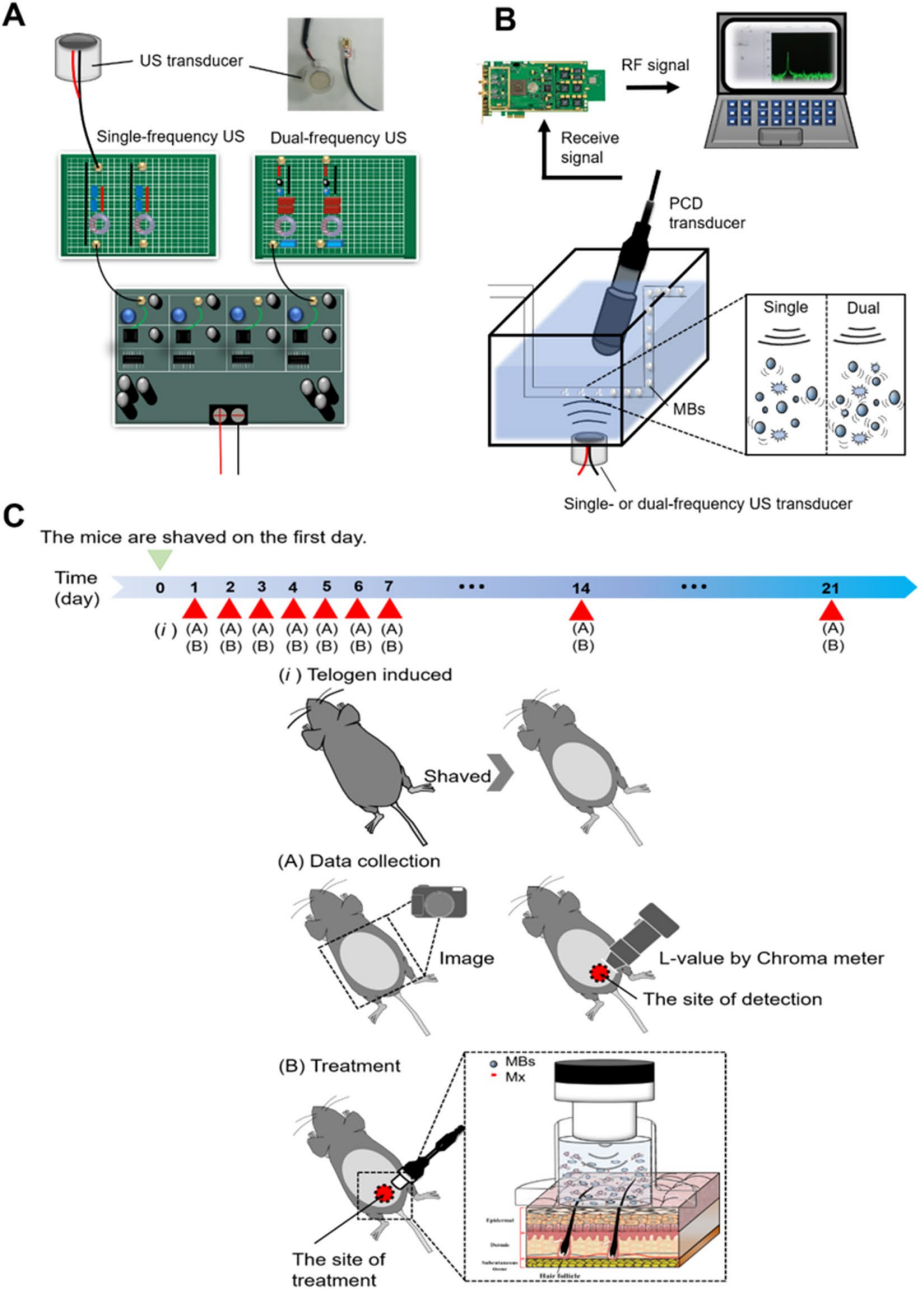
(**A**) Schematic overview of the dual-frequency US sonication system. (**B**) Schematic of the PCD setup for measuring US-mediated MB cavitation. (**C**) The scheme illustration of small animal experiment procedure in this study.

The apparatus and experimental design for the low-frequency single- and dual-frequency US sonication of a target are shown in Fig. 1A,B. A customized single- or dual-frequency US apparatus was employed in this study. The US sonication device was operated at 1 MHz and 666 kHz (1 MHz in single-frequency mode). A polyvinylidene–difluoride (PVDF) type hydrophone (Onda, Sunnyvale, California, USA; calibration range 50 kHz to 20 MHz) was mounted on a three-dimensional positioning system to measure the emission pressure distribution induced by the spherically-curved ultrasound phased arrays. The transducer was powered by a custom-designed multiple-channel driving system with bursts or continuous waves in single-frequency or dual-frequency excitation mode to produce a peak pressure of 120 kPa. The exposure pressure of this dual-frequency US transducer reached 80 kPa for both 1 MHz and 666 kHz, with a drive burst duty cycle of 20%. The transducer of a single- and dual frequency US system and a PCD were arranged confocally using a self-made holder with an included angle of approximately 120°^31^. A polyethylene (PE) tube filled with degassed water was placed in a degassed water tank 7 mm below the water surface (measured from the dual-frequency transducer to the center of the PE tube) for passive cavitation detection (PCD)^31^. The total sonication time was 240 s, and 0.5 mL of MB solution was injected at 60 s. During US sonication from 60 s to 180 s, the detector was a single-element spherically focused 1-MHz transducer with a diameter of 12.5 mm (V303-SU, Olympus, Tokyo, Japan). The transducer holder was mounted on a computer-controlled two-dimensional (2D) motion stage (HR8, Nanomotion, Yokneam, Israel). The emissions associated with MB cavitation were received by the 1-MHz transducer and then amplified by a broadband receiver (BR-640, Retec, Warwick, RI, USA). The detected emissions were then quantified using energy-spectral-density analysis with MATLAB (The MathWorks, Natick, MA, USA).

### PCD analysis

Passive cavitation emissions were received by a commercial water-immersion transducer and recorded on a personal computer using a PCI-based oscilloscope at a sampling rate of 60 MHz with 8,192 sampling points. The stable cavitation (subharmonic and harmonic components) and inertial cavitation (all accumulated energy excluding the fundamental and harmonic frequencies) were characterized, respectively. The oscilloscope was synchronized using triggers from the US sonication system. The energy spectral density (ESD; in units of V^2^·s·Hz^−1^) at frequency *ω* used to calculate the cavitation dose was defined as follows^22^:

1 $$ESD={\int }_{{\omega }_{t}-B/2}^{{\omega }_{t}+B/2}{|F(j\omega )|}^{2}{\rm{d}}\omega $$

where *F*(*jω*) is the fast Fourier transform of the passive emission signal in the time domain and *B* is the designated computation bandwidth. In our experiment designed to analyze the spectral information with respective to a specific cavitation type, we defined the integral of the stable cavitation dose (SCD) magnitude at the selected frequency including subharmonics (i.e., 0.5 × *f*_0_) and ultraharmonics (i.e., 1.5 × *f*_0_, 2.5 × *f*_0_, …), where *f*_0_ is the center frequency (i.e., 1 MHz in this case). The bandwidth was typically set to 15% of the center frequency. In contrast, the inertial cavitation dose (ICD) was calculated as the sum of the wideband-emission ESD over the entire spectrum while excluding the baseband signal (i.e., *f*_0_ = 1 MHz), subharmonics (i.e., 0.5 × *f*_0_), harmonics (i.e., 2 × *f*_0_, 3 × *f*_0_, …), and other ultraharmonics (i.e., 1.5 × *f*_0_, 2.5 × *f*_0_, …).

### Optimization of US parameters for single- and dual-frequency US-mediated MB cavitation using agarose phantoms

The sonication effects on the skin surface of applying MBs were evaluated by high-frequency US imaging using a commercial animal US imaging system (Prospect, S-Sharp Corporation, New Taipei City, Taiwan) in tissue-mimicking agarose phantoms, as described previously^32^. A 2%-agarose square-column phantom (10 mm × 20 mm × 20 mm) was constructed with a chamber (2 mm × 2 mm × 20 mm^3^) at its center to load 400 μL of 1.4 × 10^8^ MBs/mL (no dilution, group MB1), 1.4 × 10^7^ MBs/mL (10-fold dilution, group MB10), and 7 × 10^6^ MBs/mL (20-fold dilution, group MB20), followed by sonication using the single- or dual-frequency US apparatus for 1, 2, 3, and 4 min with the exposure pressure set at 80 kPa.

High-frequency US images were obtained using a transducer with a central frequency of 40 MHz, producing axial and lateral resolutions of 30 and 60 μm, respectively (Prospect, S-Sharp Corporation). The axial and lateral fields of view were 20 and 20 mm, respectively. Real-time B-mode imaging was performed, and the image planes were acquired with optimization of the gain and the time-gain compensation settings, which were kept constant throughout the experiments. Images were processed with custom MATLAB programs to evaluate the destruction efficiency^32^. The region of interest was drawn over the entire MB-loaded chamber in 2D imaging planes by the operator, the dynamic range was 50 dB, and the average pre- and postsonication image intensities were measured in B-mode images. The results obtained for the destruction efficiency were used to determine the optimal US sonication duration, and this was used in the subsequent *in vitro* and *in vivo* experiments. The MB destruction efficiency was calculated according to the image intensity using the following equation:

2 $${\rm{MB}}\,{\rm{destruction}}\,{\rm{efficiency}}( \% )=\frac{{I}_{0}-{I}_{n}}{{I}_{0}}\times 100 \% $$

where *I*_0_ is the image intensity for the original MBs and *I*_*n*_ is the image intensity of MBs for different US destruction parameters.

### Penetration depth measurements in pigskin

Fresh pigskin was obtained from the slaughterhouse affiliated to New Taipei City Meat Market. The ability to increase the penetration depth using single- or dual-frequency US-mediated MB cavitation was measured using fresh pigskin samples, with the duration from purchase to the completion of experiments being no more than 6 hours. Circular samples of pigskin from the ear were produced with a radius of 3 cm and a thickness of 3 mm. The round area of each pigskin surface were encircled with gel to prevent leakage and then loaded with model drug, Evans blue (0.1 mg; molecular weight = 960.81 Da; E2129, Sigma-Aldrich, St Louis, MO, USA) or MBs. Before US sonication, 3 mL of MBs was loaded into the treatment area of the sample and then sonication was performed successively at an exposure pressure of 80 kPa for 3 min using a single- or dual-frequency US transducer attached to the top of the sample. After removing the MBs solution from groups MB1, MB10, and MB20, 1 mL of Evans blue (0.25% w/w) was added and left for 15 min, and then the area was washed with phosphate buffered saline (PBS) three times for 1 min each.

The treated areas of pigskin were then embedded on round specimen disks (with a diameter of 2.2 cm) and placed on the –25 °C freezing stage of a cryostat (Microm HM550 series, Thermo Fisher Scientific, Bremen, Germany) for about 30 min^15,32^. The transverse sectioning was then performed at a slice thickness of 10 μm. Sections attached to the microscopy slides were air-dried at room temperature and mounted, with the distribution of the Evans blue in the cryosections determined with the aid of an upright microscope (DM 2500, Leica Microsystems). The results obtained for the penetration depth in pigskin revealed the optimal MB concentration for use with US sonication, and this was used in the subsequent *in vitro* and *in vivo* experiments.

### *In vitro* skin penetration by Mx

Static Franz diffusion cells over an area of 2.14 cm^2^ were maintained at 37 °C and used for the *in vitro* skin penetration experiments according to the procedure used in our previous study^15–17^. A 2-mm-thick fresh sample of pigskin (with the duration from purchase to the completion of experiments being no more than 6 hours) obtained from ear that included hair follicles was harvested using a Humby knife, carefully cleaned with PBS, and cut into square pieces (2 cm × 2 cm). The MB solution or 0.4 mg of Mx (molecular weight = 209.25; Sigma-Aldrich, St. Louis, MO, USA) in PBS (1 mL) (as a control) was applied to the donor cells facing the stratum corneum side of the skin, and occluded with Parafilm (Pechiney Laboratory Safety Products and Apparel, Chicago, IL, USA). The PBS (pH 7.4, 4.5 mL) was filled in receptor diffusion half cell facing the dermis side; that cell contained a magnetic stirring bar rotating at 600 rpm and 0.01% gentamicin to prevent bacterial degradation of the Mx during the penetration process. Solutions (without MBs) in the diffusion cell were filtered through a 0.22-μm micropore filter (Millex, Darmstadt, Germany)^15–17^. Aliquots (250 μL) of receptor solution were taken after various time points (0, 0.25, 0.55, 1, 2, 4, 6, 10, and 18 hours), with the cell refilled each time with the same volume of fresh receptor solution. After 0.5 hours, the 1-MHz single- or 1 MHz and 666 kHz dual-frequency US transducer was positioned 3 mm from the top of the skin to provide sonication at an exposure pressure of 80 kPa for 3 min. Samples were kept in a freezer until being analyzed using a UV-visual spectrophotometer (Lambda 40, Perkin Elmer, Bridgeville, PA, USA)^17^.

The skin sample (i.e., after 18 hours) detached from the diffusion cell was carefully rinsed five times, cut into 0.1-g pieces, and homogenized with 2 mL of receptor solution for 2 min at 10,000 rpm (Polytron-Aggregate PT3100, Kinematica, Luzern, Switzerland)^15,33^. The homogenized suspension was centrifuged for 25 min at 3,100 × *g* (Thermo Fisher Scientific), and then a UV-visual spectrophotometer was used to determine the Mx concentration in the supernatant. A sample volume of 200 μl was added to the cuvette and placed in the spectrophotometer to measure the Mx concentration. The Mx calibration curve served as the standard curve against which the absorption peaks and the corresponding concentrations of Mx in the samples were measured^15^.

### *In vivo* animal experiments

The animal experimental protocol was approved by the Institutional Animal Care and Use Committee of the National Defense Medical Center, Taipei, Taiwan. Animals were cared for in compliance with institutional guidelines and regulations (No. IACUC-13-063). The animals were housed in stainless-steel cages in an air-conditioned room with the temperature maintained at 25–28 °C and with alternating light and dark periods of 12 hours each throughout the experiments. The Six-week-old C57BL/6 mice weighing 20–25 g were obtained from Bio Lasco (Taipei, Taiwan) and divided into the following five groups (*n* = 5 per group, treatment applied once daily for 3 weeks): (i) Mx alone (group C), (ii) single-frequency US (1 MHz) and penetrating Mx (group S), (iii) dual-frequency US (1 MHz and 666 kHz) and penetrating Mx (group D), (iv) single-frequency US combined with 10-fold-diluted MBs and penetrating Mx (group S + MB10), and (v) dual-frequency US combined with 10-fold-diluted MBs and penetrating Mx (group D + MB10).

A schematic illustration of the experimental procedure was shown in Fig. 1C. An area of about 10 cm^2^ of the dorsal skin of each animal was shaved using animal clippers when the animal was 8 weeks of age, at which time all of the hair follicles were synchronized in the telogen phase, and the skin color was measured using the CR-400 Chroma Meter device (Konica Minolta Sensing, Tokyo, Japan). The single-frequency (1 MHz) or dual-frequency (1 MHz and 666 kHz) US transducer was applied at an exposure pressure of 80 kPa for 3 min, and 0.4 mg/mL Mx (corresponding to 3 mL/3.14 cm^2^) was used in all cases. The change in skin color induced by each of the treatments was assessed at predetermined times using the Chroma Meter. The luminosity index, *L*, was calculated on each measurement day before and after treatment^34^. The hair growth rate was calculated according to the *L* value using the following equation:

3 $${\rm{Hair}}\,{\rm{growth}}\,{\rm{rate}}\,( \% )=\frac{{L}_{1}-{L}_{n}}{{L}_{1}}\times 100 \% $$

where *L*_1_ is the luminosity index immediately after removing the hair and *L*_*n*_ is the luminosity index at each measurement time point.

### Histochemistry

The 8 mm × 8 mm skin tissue samples were cut from the treatment area immediately after the experiments and stored in a 10% formalin solution. Hematoxylin and eosin (Sigma-Aldrich) staining was applied, and the thickness, diameter, and number of hair follicles were analyzed using an image analysis system (TissueFAXS 3.5, TissueGnostics, Vienna, Austria) using both the scanner (TissueQuest) and cytometry (HistoQuest) analysis packages provided with that system^11^.

### Statistical analysis

The obtained data were analyzed statistically using Student’s *t*-test. A probability value of *p* < 0.05 was considered indicative of a significant difference.

## Results

### PCD of single- and dual-frequency US-mediated MB cavitation

**Figure 2:**
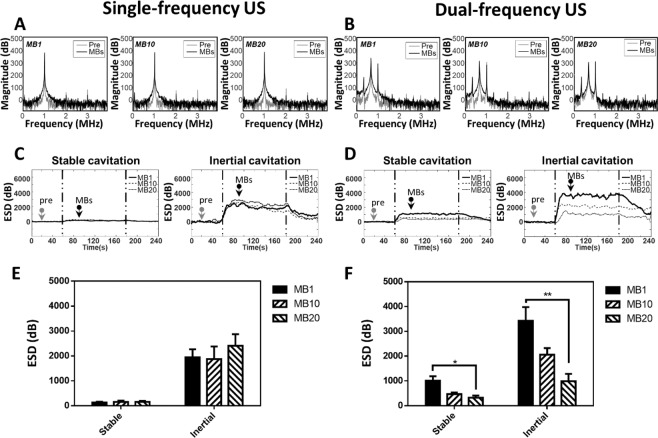
Energy spectral density in single-frequency US sonication and dual-frequency US sonication for various MB concentrations. Typical spectrum was shown in (**A,B**), which was at time point 20 sec (pre, as gray arrow) and 90 sec (MBs, as black arrow). Energy spectra in stable cavitation acquired from PCD during (**C**) single-frequency US sonication and (**D**) dual-frequency US sonication. Quantification of the ESD of SCD and ICD in panels A, C and B, D was shown in (**E**,**F)**, respectively. (*n* = 5) (*p < 0.05, **p < 0.01). Data are mean and SD values.

Figure 2 shows that cavitation occurred more easily in the dual-frequency US group than in the single-frequency group, whose design was similar to one described previously^16^. The SCD values during dual-frequency sonication were 1016.18 ± 172.13, 468.03 ± 66.55, and 323.63 ± 88.07 mV^2^·s/Hz in groups MB1, MB10, and MB20, respectively; the corresponding ICD values were 3426.92 ± 554.95, 2062.21 ± 266.54, and 989.01 ± 296.50 mV^2^·s/Hz. Both SCD and ICD during dual-frequency US sonication were correlated with the MB concentration. In comparison, the ESD during single-frequency US sonication was not reliable, with SCD values of 132.29 ± 32.76, 158.045 ± 53.07, and 159.45 ± 39.56 mV^2^·s/Hz in groups MB1, MB10, and MB20, respectively, and ICD values of 1974.28 ± 323.03, 1878.02 ± 501.73, and 2410.65 ± 464.34 mV^2^·s/Hz; the ICD was not correlated with the MB concentration. Dual-frequency US stimulation triggered more apparent stable cavitation than single-frequency US in group MB1, MB10, and MB 20. The inertial cavitation has also been enhanced during dual-frequency than single-frequency sonication in groups MB1 and MB10 (especially in group MB1). In General, dual-frequency US has been shown to significantly enhanced MB-presented acoustic cavitation effect.

### Optimization of US parameters for single- and dual-frequency US-mediated MB cavitation

**Figure 3:**
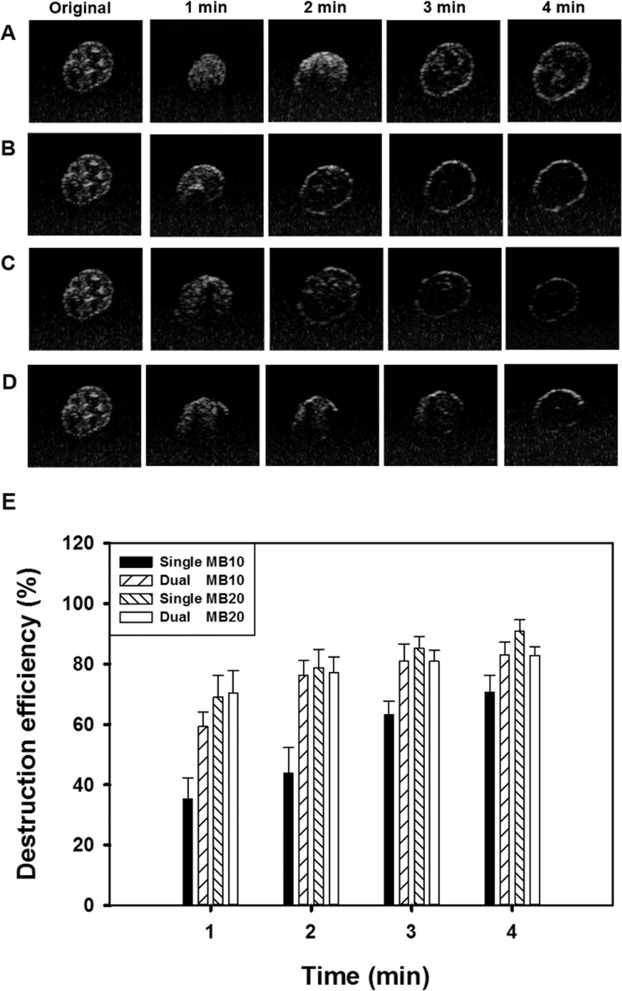
*In vitro* high-frequency US images from groups MB10 **(A,B**) and MB20 (**C,D**) before (Original) and after single- or dual-frequency US sonication for 1, 2, 3, and 4 min. (**E**) Quantification of destruction efficiency in panels A–D (n = 3 per group). Data are mean and SD values.

High-frequency US images of groups MB10 and MB20 without and with single- or dual-frequency US sonication for 1, 2, 3, and 4 min are shown in Fig. 3A–D, respectively, with the destruction efficiencies quantified in Fig. 3E. The destruction efficiencies in group MB10 for 1, 2, 3, and 4 min of sonication were 35.9%, 43.0%, 64.1%, and 71.7%, respectively, for single-frequency US sonication, and 59.1%, 74.8%, 81.3%, and 82.9% for dual-frequency US sonication. The destruction efficiencies in group MB20 for 1, 2, 3, and 4 min of sonication were 70.5%, 79.0%, 85.6%, and 88.4%, respectively, for single-frequency US sonication, and 70.7%, 77.2%, 80.9%, and 82.7% for dual-frequency US sonication. The destruction efficiencies reached to plateau for MBs at the 10-fold dilution with sonication for 3 min either in single or in dual-frequency US, and so this was used in the subsequent *in vitro* and *in vivo* experiments.

The temperature in the chamber during sonication at the different US powers increased by 1.25–1.80 °C. In this experiment, applying US at an exposure pressure of 80 kPa for 3 min almost destroyed all of the MBs in groups MB10 and MB20, and this was the highest efficiency setting in the subsequent *in vitro* and *in vivo* hair-growth experiments.

### Model drug penetration depth in pigskin with single- and dual-frequency US-mediated MB cavitation

**Figure 4:**
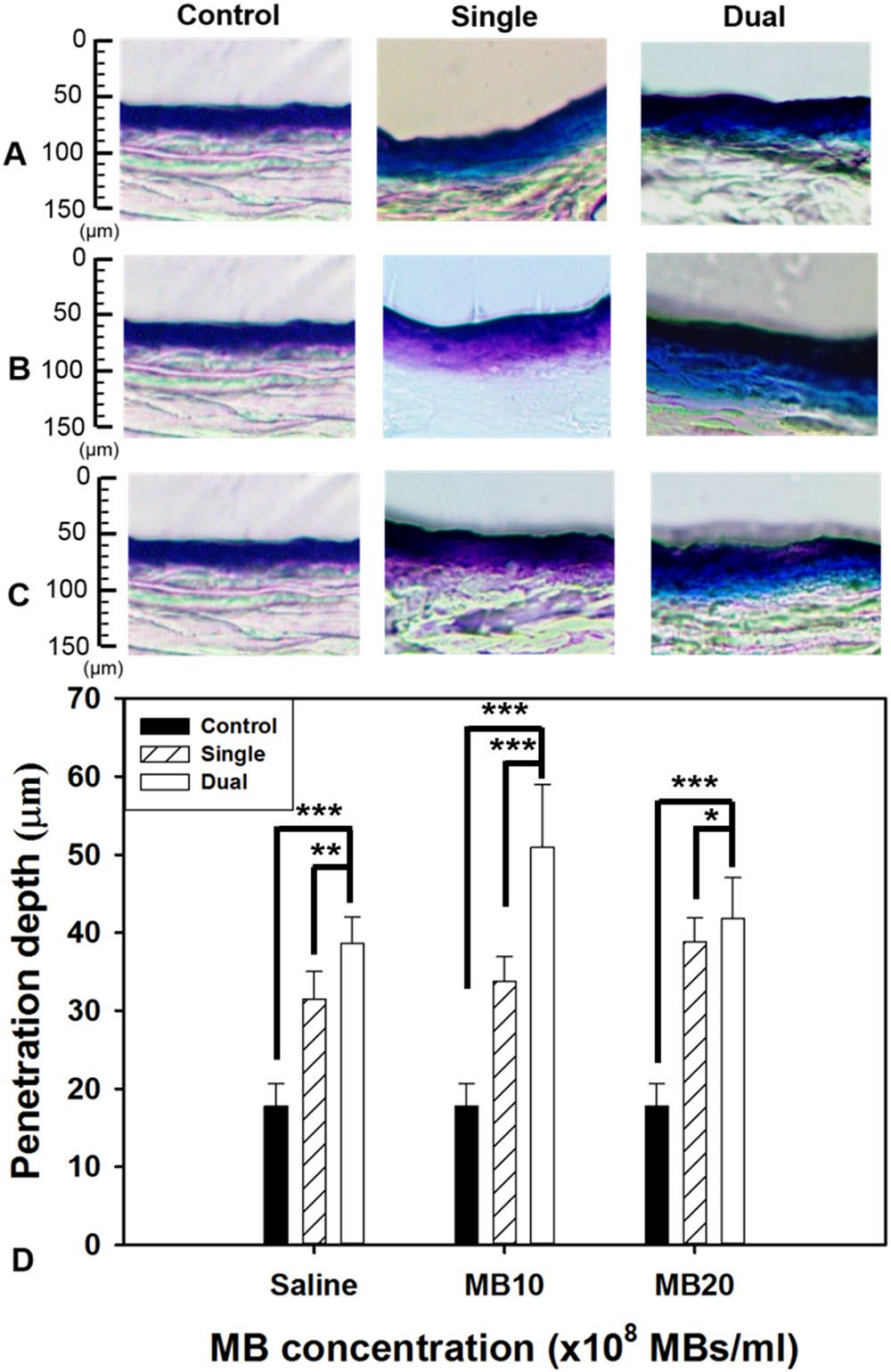
Light-microscope evaluations of drug penetration depth in pigskin samples for saline (**A**) and from groups MB10 (**B**) and MB20 (**C**) for single- or dual-frequency US sonication. (**D**) Quantification of the penetration depths of Evans blue in panels A–C (n = 5 per group) (***p < 0.001, **p < 0.01, *p < 0.05). Data are mean and SD values.

The results of the *in vitro* skin permeation studies in Fig. 4 demonstrate the ability of dual-frequency US-mediated MB cavitation in groups MB10 and MB20 to act on the hair follicle so as to facilitate Evans blue (EB) penetration as compared to single-frequency US cavitation. The pigskin samples with no treatment (group C) and after sonicating the saline (Fig. 4A) and in groups MB10 (Fig. 4B) and MB20 (Fig. 4C) with single- and dual-frequency US sonication were then cryosectioned for light-microscopy evaluation at a magnification of ×400 (Primo Star, Zeiss-Jena, Jena, Germany). Figure 4D quantifies the penetration depths in the three experimental groups (*n*=5). The degrees of penetration in both the cuticle and the epidermis were significantly greater for dual-frequency US sonication than for single-frequency US sonication, and were greatest in group MB10 (*p* < 0.001). The overall penetration depth in group C was 18.0 ± 2.36 μm, and this increased to 33.3 ± 2.87 and 51.4 ± 7.16 μm for sonication with single- and dual-frequency US in group MB10. EB penetration under single-frequency US exposure reached 38.1 ± 2.97 μm in group MB20, and was more significant than saline (30.9 ± 3.66 μm) and MB10 (33.3 ± 2.87 μm). In the contrast, EB penetration under dual-frequency US exposure reached 51.4 ± 7.16 μm in group MB10, and was more significant than saline (38.4 ± 3.00 μm) and MB20 (41.1 ± 5.11 μm). The penetration depth and uniformity both reached maximum for dual-frequency US sonication in group MB10, therefore this condition was used in the subsequent experiments involving *in vitro* skin permeation and *in vivo* animal treatments.

### *In vitro* skin permeation of Mx

**Figure 5:**
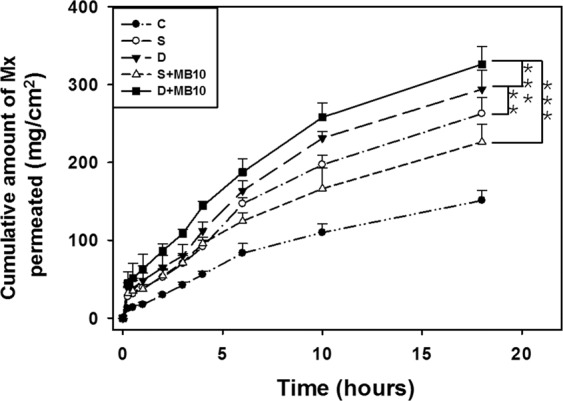
*In vitro* drug penetration through pigskin in a Franz diffusion cell at 36–37 °C in group C (Control), S (single frequency US), D (dual frequency US), S + MB10 (single frequency US combined with 10-fold dilution of microbubbles), and D + MB10 (dual frequency US combined with 10-fold dilution of microbubbles) (see Table 1) (n = 5 per group) (***p < 0.001, **p < 0.01). Data are mean and SD values.

Figure 5 shows the Mx concentrations in the five groups for percutaneous penetration over 18 hours as analyzed using the UV-visual spectrophotometer. The concentration in all groups increased rapidly during the first 6 hours and then gradually leveled off from 6 to 18 hours. At 18 hours the concentrations were significantly higher (*p* < 0.05) in group D + MB10 (81.5%) and group D (73.6%) than in groups S (65.7%), S + MB10 (56.6%), and C (37.8%). The concentration did not differ significantly (*p* > 0.05) between groups S and S + MB10. The penetration of Mx at 18 hour were 7.9% and 24.9% higher in group D + MB10 than in groups D and S + MB10, respectively. The transdermal Mx penetration was significantly higher in group D than in group S (*p* < 0.01), and in group D + MB10 than in groups D (*p* < 0.001) and S + MB10 (*p* < 0.001), respectively. Table 1 indicates that the amount of Mx deposited in the skin was significantly greater in group C than in the other four groups at 18 hours. The total amount of Mx that penetrated was significantly greater in group D + MB10 than in the other four groups.

**Table 1:** Total amount of Mx that permeated after 18 hours (deposited on the skin plus penetrated across the skin).

Parameter	Skin weight (g)	Amount of Mx deposited on skin (μg/cm^2^)	Amount of Mx penetrated across skin (μg/cm^2^)	Total amount of Mx permeated (μg/cm^2^)
Group				
C	0.157 ± 0.026	75.41 ± 2.09 (18.9%)	151.25 ± 10.20 (37.8%)	226.65 ± 12.29 (56.7%)
S	0.168 ± 0.032	56.86 ± 3.33 (14.2%)	262.71 ± 17.10 (65.7%)	319.57 ± 20.43 (79.9%)
S + MB10	0.171 ± 0.033	62.81 ± 3.13 (15.7%)	226.40 ± 18.77 (56.6%)	289.21 ± 21.90 (72.3%)
D	0.143 ± 0.021	50.08 ± 1.12 (12.5%)	294.21 ± 19.81 (73.6%)	344.29 ± 20.93 (86.1%)
D + MB10	0.131 ± 0.026	45.77 ± 2.15 (11.4%)	326.19 ± 18.63 (81.5%)	371.97 ± 20.78 (92.9%)

Data are mean ± SD values.

### *In vivo* hair growth enhancement with MB-enhanced dual-frequency US sonication

**Figure 6:**
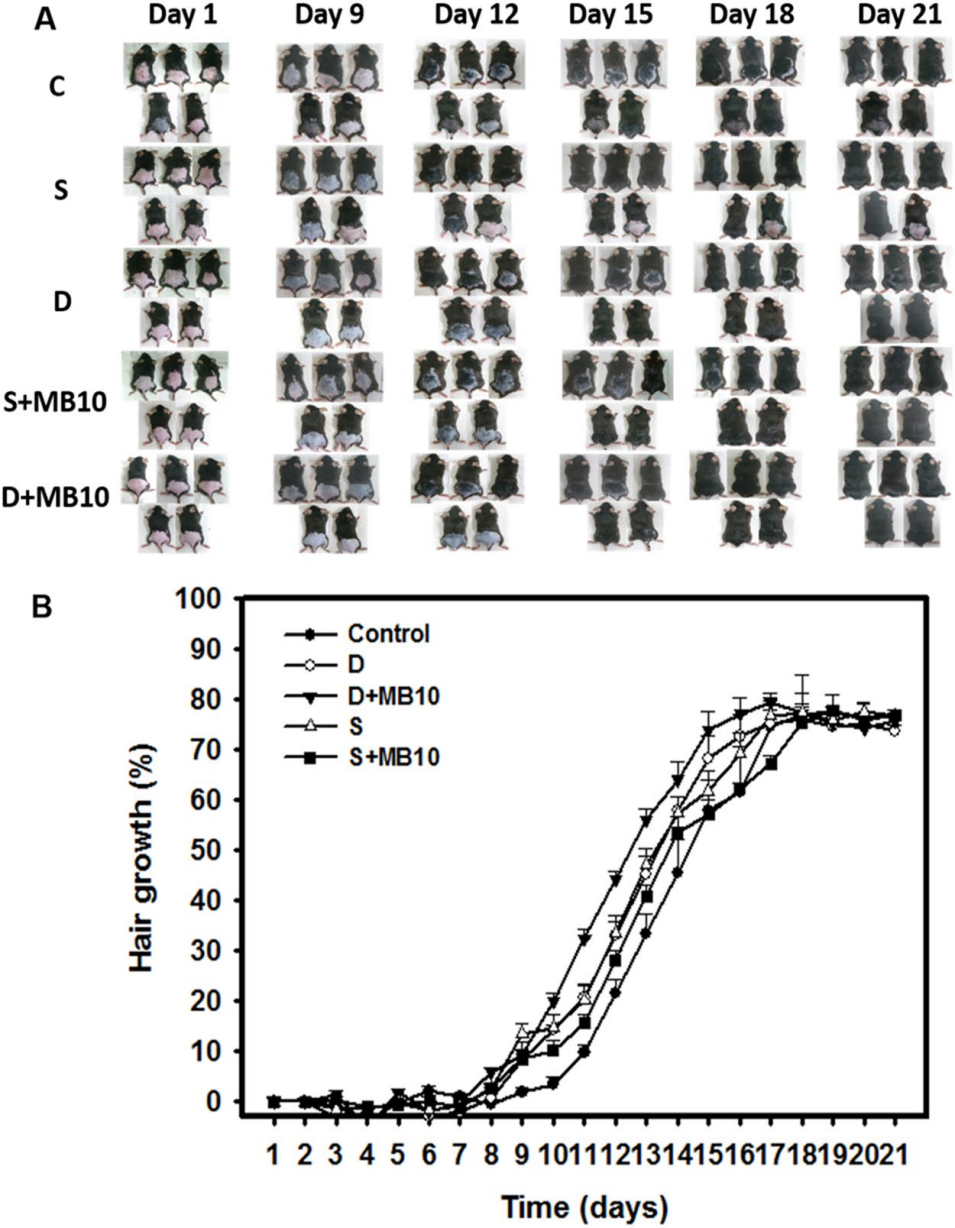
*In vivo* hair growth enhancement with MB-enhanced single- or dual-frequency US sonication. (**A**) Gross observations of the dorsal skin of C57BL/6 mice. The dorsal skin surfaces of the mice were shaved, and then test compounds and treatments were topically applied in group C (Control), S (single frequency US), D (dual frequency US), S + MB10 (single frequency US combined with 10-fold dilution of microbubbles), and D + MB10 (dual frequency US combined with 10-fold dilution of microbubbles) for 3 weeks. (**B**) Quantification of hair growth rates on the dorsal skin after shaving the hair in various mouse groups over 21 days. Data are mean and SD values (n = 5).

Figure 6A shows photographs of mouse skin in an untreated animal (day 1) and in groups C, S, D, S + MB10, and D + MB10 at various time points after treatment. At day 9, the skin brightness for the five mice in group D + MB10 was more effectively decreased compared to groups S + MB10 (decreased in four mice), D (decreased in four mice), S (decreased in four mouse), and C (decreased in two mice). At day 12, the hair growth was greater in all mice in group D + MB10 than in the other four groups.

Figure 6B demonstrates the effects of Mx on dorsal hair growth over 21 days. At days 11 and 14, the growth rates in group D + MB10 had increased by 32.4% and 64.2%, respectively. At day 11 there were obvious significant differences (*p* < 0.05) between group D + MB10 and the other four groups. At day 16, the growth rate had reached a plateau in group D + MB10, with an increase of 77.1%, while the growth rates in groups C, S, D, and S + MB10 had increased by 58.6%, 69.3%, 72.6%, and 64.3%, respectively. At that time point the growth rate did not differ significantly between groups D + MB10 and D (*p* > 0.05).

**Figure 7:**
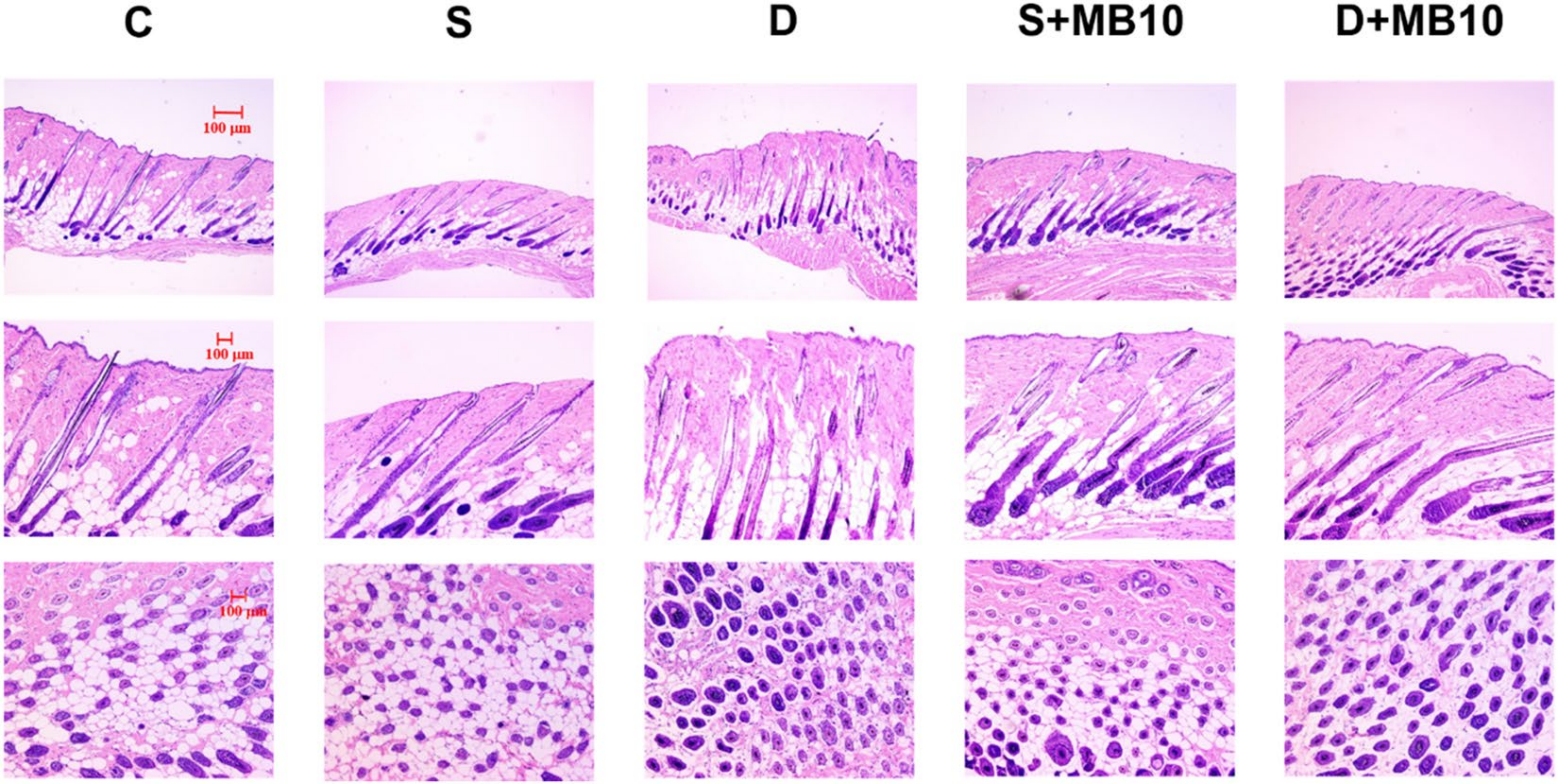
Histological observations of hair follicles in mice skin after 21 days in group C (Control), S (single frequency US), D (dual frequency US), S + MB10 (single frequency US combined with 10-fold dilution of microbubbles), and D + MB10 (dual frequency US combined with 10-fold dilution of microbubbles). Vertical (top and middle panels) and coronal (bottom panels) views of the hair follicles.

**Figure 8:**
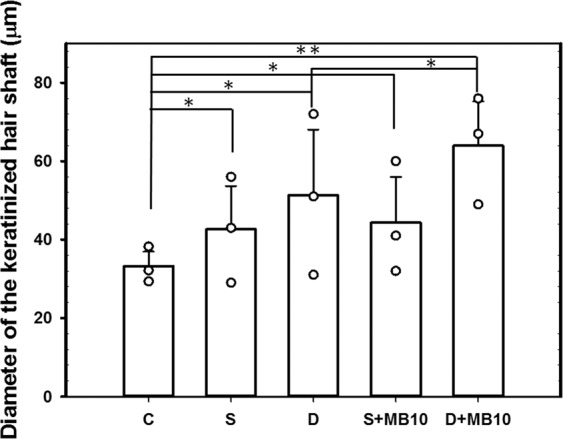
Histological quantification of the diameter of the keratinized hair shaft after 21 days in groups C, S, S + MB10, D, and D + MB10 (n = 3) (**p < 0.01, *p < 0.05). Data are mean and SD values.

The histology images in Fig. 7 indicate that no skin damage was evident in any of the US sonication groups. Histological analysis of transverse and coronal sections (Fig. 7) revealed significant increases in the diameter (Fig. 8) of keratinized hair shafts after 21 days in groups S, S + MB10, D, and D + MB10. The enhancements of keratinized hair shafts were greatest in groups D and D + MB10, and was more significantly increased in group D + MB10 than in group D (*p* < 0.05). Moreover, the number of hair follicles in groups D and D + MB10 was increased after treatment in Fig. 7.

## Discussion

Dual-frequency US sonication of MBs induces larger cavitation-related emissions due to radial oscillations occurring at higher harmonics^35^. Dual-frequency excitation is known to be an efficient method for enhancing the cavitation of MBs in sonography as well as for minimizing any associated unwanted damage^35^. Figure 2 shows that the SCD values for various concentrations of MBs were more evident in dual-frequency US sonication, and this is consistent with theoretical expectations; however, during single-frequency sonication, the cavitation dose did not provide a useful index for the MB concentration. This confirms that using multifrequency US sonication improves the utilization of MBs for transdermal applications.

This study demonstrated that both stable and inertial cavitation can be enhanced during dual-frequency sonication under the presence of microbubbles. We observed that dual-frequency US at an appropriate intensity provided higher stable cavitation activity than single-frequency US, which can be utilized to stimulate cell membrane mechanochannels without inducing cell damage^22,26,27^. Cavitation was found to be proportional to MBs concentration in dual-frequency US stimulation group, but not in single-frequency US group. During the PCD test, we estimated that cavitation lasted for about 3 and 4 min during single- and dual-frequency US sonication, respectively, at 80 kPa in groups MB10 and MB20. In high-frequency US imaging, the destruction of MBs was compared with the penetration depths measured in pigskin. The penetration depth is related to the destruction efficacy of MBs, and this was greatest in group MB10 for 3 min of dual-frequency US sonication. Therefore, this exposure condition was used in the subsequent *in vitro* and *in vivo* hair-growth experiments.

Our previous *in vitro* profiles for the permeation of Mx through skin samples mediated by 140 kPa US (3 W/cm^2^ for 1 min; ST2000V, Nepagene, Ichikawa, Japan) indicate that the concentrations increased more rapidly in the group that combined US sonication and MBs and included Mx separately than in the other groups during the first 6 hours, and then became closer to or even lower than those in the group with US sonication and Mx-coated MBs from 6 to 18 hours^17^. The present study results indicate that combining single-frequency US sonication with MBs increased the permeation more than that in the control group at all measurement time points. However, the permeated concentration did not differ between single-frequency US sonication alone and single-frequency US sonication combined with MBs at first 4 hours. This might have been due to single-frequency US sonication at 80 kPa not destroying MBs completely and thereby reducing the efficacy of inertial cavitation by MBs. Under the same 80-kPa acoustic pressure, dual-frequency US sonication and dual-frequency US-mediated MB cavitation increased the permeation more rapidly relative to the other groups throughout the 18-hour experiments, with this being greater when MBs were included. The results indicated that dual-frequency US sonication induced greater inertial cavitation and is a more efficient method than single-frequency US sonication, and so allows the use of lower and hence safer acoustic pressures. Moreover, dual-frequency US sonication combined with MB cavitation enhanced TDD more significantly compared to using dual-frequency US sonication alone.

Topical Mx (Rogaine) is approved for the treatment of androgenetic alopecia (AGA) and the only medication that can be used by both men and women^36^. AGA is the most common cause of hair loss and is induced by androgens in genetically susceptible hair follicles. In such hair follicles dihydrotestosterone binds to the androgen receptor and this hormone–receptor complex then activates the genes that control the gradual transformation of large, terminal follicles into small, miniaturized follicles^17,37^. However, Mx does not work on completely bald areas and only works over the long term if it is used continuously. In the present study, we observed that *in vivo* hair growth enhancement with Mx was greater in group D + MB10 than in the other groups. The hair growth rate from 9 to 14 days did not differ significantly among groups D, S, and S + MB10. However, the histology (Fig. 7) confirmed that the diameters of keratinized hair shafts after 21 days were significantly increased in groups D and D + MB10 compared to in groups S and S + MB10 (Fig. 8). There was greater variability for dual-frequency US sonication alone than for dual-frequency US sonication combined with MBs. We therefore assume that the addition of MBs facilitates the stability of dual-frequency US sonication for hair growth enhancement.

## Conclusion

This study investigated a novel dual-frequency US sonication technique for increasing the efficacy of Mx in enhancing hair growth. Combining dual-frequency US sonication with MBs can enhance cavitation more significantly during dual-frequency sonication than single-frequency sonication in higher concentration of MBs. This method enhances both the deposition of Mx into the skin and the penetration of Mx across the skin *in vitro*, and increase hair growth than in the other groups at most of the measurement time points in *in vivo* small-animal study. Histology images revealed that dual-frequency US sonication can increase the diameter of keratinized hair shafts, and combining dual-frequency US with MBs can improve the variability in hair growth enhancement.
